# Independent Multi-Cohort RNA-Seq Analysis Does Not Support Recurrent DNAJB1::PRKACA Fusion in Hepatoblastoma or Biliary Atresia. Comment on Fleifil et al. DNAJB1-PKAc Kinase Is Expressed in Young Patients with Pediatric Liver Cancers and Enhances Carcinogenic Pathways. *Cancers* 2025, *17*, 83

**DOI:** 10.3390/cancers18162681

**Published:** 2026-08-19

**Authors:** Fahreddin Palaz, Xuanxuan Yu, Julia Bruner, Michael Feely, Guoshuai Cai, Sergio Duarte, Ali Zarrinpar

**Affiliations:** 1 Department of Surgery, College of Medicine, University of Florida, Gainesville, FL 32610, USA; fahreddin.palaz@surgery.ufl.edu (F.P.); xuanxuan.yu@surgery.ufl.edu (X.Y.); juliabruner@ufl.edu (J.B.); guoshuai.cai@surgery.ufl.edu (G.C.); sergio.duarte@surgery.ufl.edu (S.D.); 2 Department of Biostatistics, College of Public Health and Health Sciences, University of Florida, Gainesville, FL 32610, USA; 3 Department of Pathology, Immunology and Laboratory Medicine, College of Medicine, University of Florida, Gainesville, FL 32610, USA; mfeely@ufl.edu

We read with great interest the article by Fleifil et al. in *Cancers*, reporting DNAJB1::PRKACA fusion expression in approximately 70% of hepatoblastoma/hepatocellular neoplasm-not otherwise specified (HBL/HCN-NOS) samples and in 3 of 6 biliary atresia (BA) samples [1]. While our manuscript was in preparation, a separate thoughtful correspondence by Arif et al., as well as the considerate reply by Fleifil et al., were also published [2,3]. The Arif consortium evaluated the *DNAJB1::PRKACA* fusion using multiple independent and complementary approaches at the RNA and DNA levels, including bulk RNA-seq-based fusion analyses, RT-PCR-based assays with Sanger confirmation, targeted RNA fusion testing with Archer FusionPlex, and whole-genome sequencing with structural variant detection. Across 225 independent experiments in 148 hepatoblastoma samples, all approaches uniformly failed to detect the fusion, underscoring the robustness of their negative findings. Their essential discussion centered on the expression of *DNAJB1::PRKACA* fusion in HBL cases. Given the major biological and diagnostic implications of the findings reported by Fleifil et al. for both fibrolamellar carcinoma (FLC) and non-fibrolamellar liver diseases, we believe that rigorous independent validation is essential.

***Reported fusion-protein abundance and associated biological effects in HBL/HCN-NOS are difficult to reconcile with the proposed need for ultrasensitive methods for fusion transcript detection***

In the original study, the authors reported fusion-protein expression in approximately 70% of HBL/HCN-NOS cases, including 14 samples that they classified as having high fusion expression [1]. This conclusion was based on immunohistochemistry (IHC) and Western blot assays. In several HBL samples, including #49, #72, #74, #97, #114, and #116, the intensity of putative DNAJ-PKAc fusion bands on Western blots appears comparable to or greater than that of the wild-type PKAc band. In some instances, putative DNAJ-PKAc band intensities in HBL also appear comparable to those seen in FLC samples on the same blot. A similar pattern is evident in samples obtained from BA patients, where 3 of 6 reported samples (patients #2, #4, and #5) show a fusion-band intensity comparable to or stronger than wild-type PKAc. Accordingly, the original study presents DNAJB1::PRKACA fusion in HBL/HCN-NOS as a highly frequent event associated with proposed biological consequences, including increased proliferation, fibrosis with lamellar-like features, repression of tumor suppressors, and FLC-like molecular alterations [1]. In their reply to Arif et al., Fleifil et al. suggest that detection of *DNAJB1::PRKACA* in non-FLC samples may require an assay with unusually high sensitivity and may be missed by methods optimized for the stronger fusion expression typically observed in FLC [3]. However, it is difficult to reconcile the Western blot patterns presented in the original study by Fleifil et al. and the multiple biological associations attributed to DNAJB1::PRKACA fusion positivity with the view that the uniformly negative findings obtained by Arif et al. in an international five-institution analysis of 148 HBL samples, involving 225 independent experiments and multiple sensitive and orthogonal DNA- and RNA-based methods, can be primarily explained by insufficient assay sensitivity or by low fusion expression.

It should be noted that protein-level assays such as IHC or Western blot can generate apparent positive signals even when transcript-level evidence is absent because antibodies may cross-react with the wild-type PKAc or other unrelated proteins, the fusion protein can be unusually stable or highly expressed from a low-abundance transcript, and post-translational modifications or proteolytic processing can create epitopes that are recognized despite the corresponding mRNA being below the detection limit of sequencing-based methods. However, such mechanisms might explain isolated protein–RNA discordance, particularly in cases with weak putative fusion-kinase signals, but they do not readily account for the broader reported pattern of fusion positivity in approximately 70% of HBL/HCN-NOS cases and 3 of 6 BA samples, including multiple specimens with putative DNAJ-PKAc bands comparable to or stronger than wild-type PKAc. No evidence has been presented for enhanced translation, exceptional fusion-protein stability, or disease-specific post-translational processing in HBL/HCN-NOS or BA. Moreover, qRT-PCR with amplification products visualized by gel electrophoresis detected the fusion transcript in HBL samples #72 and #97, both of which showed strong putative DNAJ-PKAc expression, whereas no fusion amplicon was detected in sample #76 despite one of the strongest putative DNAJ-PKAc bands, highlighting unresolved discordance within the original dataset (Figure 1 of Ref. [1]). In contrast, the Arif consortium reported 0/18 HBL cases by RT-PCR–Sanger sequencing, 0/7 by RT-PCR across three spatially distinct tumor regions, 0/11 by dual RT-PCR, and 0/10 by Archer FusionPlex, together with uniformly negative RNA-seq- and whole-genome sequencing-based analyses [2]. Given that conventional qRT-PCR detected the fusion in HBL samples with strong bands attributed to DNAJ-PKAc, the uniformly negative results across substantially larger independent cohorts examined using multiple sensitive assays are difficult to attribute solely to inadequate assay sensitivity or low fusion abundance.

Riggle et al. found that total PKA-C protein abundance, comprising DNAJ-PKAc and wild-type PKAc, was approximately 2.6-fold higher in FLC tumors than wild-type PKAc in paired non-tumor liver, whereas, within FLC tumors, *DNAJB1::PRKACA* mRNA was approximately 11-fold more abundant than wild-type PRKACA mRNA [4]. Although these measurements do not establish a fixed quantitative transcript–protein relationship, they show that prominent DNAJ-PKAc expression in bona fide FLC occurs in the setting of abundant, rather than trace-level, fusion transcript. Consistently, junction-targeting shRNA or siRNA produced progressive depletion of DNAJ-PKAc in cellular and FLC patient-derived xenograft models while largely sparing wild-type PRKACA, demonstrating the continued dependence of fusion-protein expression on the *DNAJB1::PRKACA* transcript [5,6]. This molecular context cannot be assumed to apply unchanged to HBL or BA, as post-transcriptional regulation and protein turnover may differ across disease states. Nevertheless, no HBL- or BA-specific mechanism has been demonstrated that would reconcile putative DNAJ-PKAc levels comparable to or exceeding wild-type PKAc with consistently weak or absent fusion-transcript evidence. This discordance is particularly notable given the broad FLC-like molecular, transcriptomic, histopathological, and functional features attributed to putative fusion-positive HBL/HCN-NOS by Fleifil et al. [1]. Taken together, these observations make it difficult to attribute the repeated failure to detect *DNAJB1::PRKACA* across independent HBL/HCN-NOS and BA cohorts primarily to low fusion-transcript abundance or inadequate assay sensitivity.

Accordingly, if multiple HBL or BA specimens expressed DNAJ-PKAc at levels comparable to or exceeding wild-type PKAc, with some reaching levels similar to those observed in FLC, at least some corresponding fusion-junction reads would reasonably be expected in RNA-seq libraries in which reads spanning the PRKACA exon 1–2 junction were readily recoverable. We therefore evaluated publicly available FLC, HBL, and BA RNA-seq datasets using two complementary approaches: standard fusion calling and direct within-sample interrogation of wild-type and fusion-junction reads.

***Independent multi-cohort RNA-seq analyses do not support DNAJB1::PRKACA as a recurrent event in hepatoblastoma or biliary atresia***

The detection of DNAJ-PKAc fusion protein in the livers of HBL and BA patients is surprising and would have significant diagnostic and biological implications. Naturally, as it did for the authors of Arif et al., this report triggered our curiosity, and we sought to examine it directly by analyzing four independent HBL RNA-seq datasets using Arriba [7], an established fusion-calling tool for RNA-seq data. The overall dataset comprised 78 samples from cohorts across multiple geographic regions and institutions, including the United States (Children’s Hospital of Pittsburgh, GSE89775 [8]), France (University of Bordeaux, GSE104766 [9]), Germany (Ludwig-Maximilians-University Munich, GSE151347 [10]), and Spain (Germans Trias i Pujol Research Institute, GSE133039 [11]). Interestingly, our detailed analysis did not detect a *DNAJB1::PRKACA* fusion transcript in any of the 78 HBL samples (Table 1), in clear contrast to the original report.

The authors also propose that both patients’ ages at the time of fusion analysis and differences between demographic regions may explain the contrast between their original findings and the findings of Arif et al. [3]. Therefore, we extended our analysis to additional RNA-seq datasets relevant to this clinical context, including 23 HBL samples from Cincinnati Children’s Hospital Medical Center (CCHMC) (GSE81928 [12]) and 171 pediatric BA samples from the multicenter Childhood Liver Disease Research Network (ChiLDReN), including infants evaluated at CCHMC (GSE122340 [13]), which, to our knowledge, represents the largest publicly available transcriptional cohort in BA. In the HBL cohort, 20 of 23 samples were from patients younger than 5 years of age, and BA patients were younger than 1 year. Across both cohorts, we found no evidence of a *DNAJB1::PRKACA* fusion transcript in any sample (Table 1). Although these may be distinct patients from those included in the original study by Fleifil et al., the ages at sampling were comparable, and both cohorts included representation from CCHMC.

To confirm that the pipeline could recover the canonical *DNAJB1::PRKACA*, we applied the same approach to three independent publicly available FLC RNA-seq cohorts comprising 25 liver tumor samples, derived from Memorial Sloan Kettering Cancer Center (MSKCC) (GSE63018 [14]), Cornell University-Fibrolamellar Cancer Foundation (FCF)-associated cohort (GSE181922 [15]), and Fred Hutchinson Cancer Center (FHCC) (GSE250059 [16]). In all 25 FLC cases, Arriba identified the canonical FLC *DNAJB1::PRKACA* fusion as a high-confidence, in-frame event, with a recurrent breakpoint corresponding to the DNAJB1 exon 1-PRKACA exon 2 junction. In contrast, *DNAJB1::PRKACA* was not detected in any of the analyzed HBL or BA RNA-seq cohorts (Table 1). It should be noted that sequencing yields were not uniform across cohorts, with the Cornell-FCF FLC cohort showing higher sample-level yields than most HBL and BA cohorts. Nevertheless, the sample-level sequencing-yield ranges of the MSKCC and FHCC FLC cohorts overlapped those of multiple paired-end HBL and BA cohorts.

RNA-seq-based fusion detection has long been performed using paired-end reads in the 50–100 bp range, and widely used fusion callers were developed and validated on datasets within this range [17,18,19]. In our analysis, Arriba detected the canonical *DNAJB1::PRKACA* fusion in all 25 FLC samples across cohorts with read lengths of 50, 76–77, and 150 bp. In contrast, no fusion was detected in any HBL or BA sample, including paired-end cohorts with read lengths of 75–125 bp and sequencing yields overlapping those of fusion-positive FLC cohorts. Thus, the analyzed HBL and BA libraries fell within read-length ranges widely used for fusion detection and overlapped the fusion-positive FLC datasets in both read length and sequencing output, supporting their technical suitability for fusion-calling analysis. Accordingly, differences in read length do not readily account for the complete absence of *DNAJB1::PRKACA* calls across the analyzed HBL and BA cohorts.

The uniformly negative findings are also difficult to attribute to interpatient or intratumoral heterogeneity. Fleifil et al. reported frequent fusion positivity from routinely sampled specimens without presenting spatially resolved analyses, showing that fusion-positive cells were confined to rare tumor regions or minor subclones [1]. At the reported prevalence, at least some positive specimens would be expected across the large independent cohorts examined by Arif et al. and in our study, even if fusion expression varied among patients or tumor regions. Moreover, the Yarchoan group within the Arif consortium examined three spatially distinct regions from each of seven HBL tumors by RT-PCR and found all regions negative [2]. Taken together, these findings make interpatient or intratumoral heterogeneity unlikely explanations for the uniformly negative results across these independent studies.

***Direct within-sample interrogation of RNA-seq reads does not support DNAJB1::PRKACA as a recurrent event in hepatoblastoma or biliary atresia***

The fusion-calling analysis asked whether an event reported in approximately 70% of HBL/HCN-NOS tumors and 3 of 6 BA samples, often with substantial putative DNAJ-PKAc expression, would generate at least some calls across multiple independent datasets. Because standard callers readily detected the canonical fusion in FLC, its complete absence in HBL and BA prompted direct interrogation of the underlying RNA-seq reads for fusion-junction evidence. To trace the fusion transcript directly, we searched each RNA-seq library in both forward and reverse-complement orientations for matched exact 20-nucleotide sequences spanning either the canonical DNAJB1 exon 1–PRKACA exon 2 junction (GGGGAGGAAGTGAAAGAATT) or the wild-type PRKACA exon 1–2 junction (CAGGAGAGCGTGAAAGAATT). Each query contained 10 nucleotides on either side of the junction, providing a 10-nucleotide anchor for each side, consistent with the minimum anchor length used for split-read support [20]. The downstream 10 nucleotides were identical and derived from PRKACA exon 2, whereas the upstream 10 nucleotides derived from DNAJB1 exon 1 or PRKACA exon 1. Recovered reads were examined in full and aligned to the corresponding *DNAJB1::PRKACA* or PRKACA cDNA reference to confirm bona fide junction-spanning matches.

This design provided two complementary forms of internal validation. First, recovery of the PRKACA exon 1–2 junction confirmed that bona fide PRKACA-derived junction reads were present and retrievable from the same libraries. Because matched PRKACA and fusion queries were applied within each library, library preparation, read length, sequencing depth, and overall output were shared and therefore could not explain selective recovery of the PRKACA exon 1–2 junction, although equal transcript abundance or local junction coverage was not assumed. Second, PRKACA provided a biologically informative reference. In bona fide FLC, prominent DNAJ-PKAc expression occurs in a transcriptomic context in which *DNAJB1::PRKACA* mRNA markedly exceeds wild-type PRKACA mRNA [4]. Thus, even without assuming the same quantitative relationship in HBL/HCN-NOS or BA, the many putative fusion-positive cases reported to express DNAJ-PKAc at levels comparable to or greater than PKAc would reasonably be expected to include at least some with detectable fusion-junction reads, particularly when PRKACA junction reads were readily recoverable from the same library.

Using the 20-nucleotide queries, bona fide PRKACA exon 1–2 junction reads were recovered from the vast majority of HBL and BA libraries, confirming that informative PRKACA junction-spanning reads were present in most datasets. The native junction was not recovered in a small minority of samples, several of which had low overall sequencing output and were therefore less informative. In the FLC libraries, the identical query approach readily recovered both the PRKACA exon 1–2 junction and the canonical DNAJB1 exon 1–PRKACA exon 2 fusion junction, demonstrating recovery of both junctions in FLC. Because this exact-sequence search targeted the canonical DNAJB1 exon 1–PRKACA exon 2 junction, it would not detect alternative *DNAJB1::PRKACA* transcript isoforms involving different exon junctions, such as the minority chimera reported in a subset of FLC cases [21]. In contrast, although the PRKACA junction was widely recovered in HBL and BA, no canonical *DNAJB1::PRKACA* junction read was identified in any sample. This absence of fusion-junction evidence, despite successful recovery of both junctions in FLC and widespread detection of PRKACA exon 1–2 junction in HBL and BA, is difficult to reconcile with a recurrent fusion reported in approximately 70% of HBL/HCN-NOS and 3 of 6 BA cases, particularly given that putative DNAJ-PKAc levels were comparable to or exceeded PKAc in a substantial number of reported samples and, in some cases, approached those observed in FLC.

## Conclusions

Taken together, the discrepancy between the recurrent detection of DNAJB1::PRKACA in HBL and BA reported by Fleifil et al. and the uniformly negative findings across larger independent cohorts is not adequately explained by differences in assay sensitivity, patient age, geographic background, sequencing characteristics, or interpatient and intratumoral heterogeneity. Moreover, the proposed recurrence of the fusion, the substantial putative DNAJ-PKAc bands observed in many specimens, and the FLC-like molecular, transcriptomic, histopathological, and functional alterations attributed to putative fusion-positive HBL/HCN-NOS are difficult to reconcile with the assertion that the fusion transcript requires unusually sensitive assays for detection. In our analysis of multiple independent HBL and BA RNA-seq cohorts, each library was evaluated individually by both Arriba-based fusion calling and direct interrogation of the canonical DNAJB1 exon 1–PRKACA exon 2 junction sequence, and neither approach provided evidence that *DNAJB1::PRKACA* is a recurrent event. This is consistent with Arif et al., whose international consortium across five institutions found no fusion in 148 HBL samples evaluated through 225 independent assays using multiple complementary DNA- and RNA-based approaches, including highly sensitive targeted methods [2].

Given the potential biological and clinical implications, we believe that a substantial number of the originally reported “fusion-positive” non-FLC cases should be directly re-examined using multiple orthogonal approaches at both the RNA and DNA levels, including junction-spanning RT-PCR followed by Sanger sequencing, independent fusion analysis of RNA-seq data, and, where feasible, genomic structural variant analysis. Such validation would require appropriate controls to exclude contamination and other technical artifacts, detailed reporting of the methods and results, and confirmation in independent cohorts. Misclassification of HBL or BA as DNAJB1::PRKACA-positive could erroneously direct patients toward therapeutic strategies that are currently recommended for FLC, potentially exposing them to ineffective treatment and unnecessary toxicity [22]. Pending such additional validation, the interpretation of antibody-mediated detection of DNAJB1::PRKACA expression in a recurrent subset of HBL/HCN-NOS or BA cases should merit careful consideration.

## Figures and Tables

**Table 1:** Sequencing characteristics and *DNAJB1::PRKACA* fusion detection by Arriba across independent HBL, BA, and FLC RNA-seq cohorts.

Disease Group	GEO Series	Institution/Cohort	*n* Samples Analyzed	Platform	Layout *	Median Sequencing Yield per Sample (Gb)	Range (Gb)	Read Length ***	*DNAJB1::PRKACA* Detected by Arriba	Study
HBL	GSE89775	Children’s Hospital of Pittsburgh	10	Illumina HiSeq 2500	PE	7.93	4.64–9.39	100	0/10	[8]
HBL	GSE104766	University of Bordeaux	25	Illumina HiSeq 2500	PE	11.73	1.38–17.78	125	0/25	[9]
HBL	GSE151347	LMU Munich	11	Illumina HiSeq 2000	PE	8.67	4.35–11.39	101	0/11	[10]
HBL	GSE133039	Germans Trias i Pujol Research Institute	32	Illumina HiSeq 2500	Mixed (31 PE, 1 SE) **	5.49	1.11–9.71	100	0/32	[11]
HBL	GSE81928	CCHMC	23	Illumina HiSeq 2500	PE	4.48	2.15–15.55	75	0/23	[12]
BA	GSE122340	ChiLDReN-CCHMC	50	Illumina HiSeq 2500	PE	7.44	5.35–13.07	124–125	0/50	[13]
BA	GSE122340	ChiLDReN-CCHMC	121	Illumina HiSeq 2500	SE	0.84	0.27–3.10	50	0/121	[13]
FLC	GSE63018	MSKCC	10	Illumina HiSeq 2500	PE	7.52	5.20–10.57	50	10/10	[14]
FLC	GSE181922	Cornell-FCF	7	Illumina NextSeq 500	PE	19.31	16.23–24.75	76–77	7/7	[15]
FLC	GSE250059	FHCC	8	Illumina HiSeq 2500	PE	13.93	9.05–17.62	150	8/8	[16]

* PE: Paired-end; SE: Single-end; ** GSE133039 included 31 paired-end libraries and 1 single-end library (SRR9329005). *** Read length refers to the length of each read for PE libraries.

## Data Availability

The RNA-seq datasets analyzed in this study are publicly available through the NCBI Gene Expression Omnibus (GEO) under the accession numbers provided in Table 1. No new sequencing data were generated in this study.
